# Three novel *ATG16L1* mutations in a patient with acute myocardial infarction and coronary artery ectasia: A case report

**DOI:** 10.1097/MD.0000000000024497

**Published:** 2021-01-29

**Authors:** Falan Han, Bo Yan

**Affiliations:** a Cheeloo College of Medicine, Shandong University, Jinan; b Shandong Provincial Key Laboratory of Cardiac Disease Diagnosis and Treatment; c The Center for Molecular Genetics of Cardiovascular Diseases; d Shandong Provincial Sino-US Cooperation Research Center for Translational Medicine, Affiliated Hospital of Jining Medical University, Jining Medical University, Jining, Shandong, China.

**Keywords:** acute myocardial infarction, ATG16L1, coronary artery ectasia, gene mutations, single nucleotide polymorphisms

## Abstract

**Introduction:**

Acute myocardial infarction (AMI) is a specific type of coronary artery disease (CAD) caused by the rupture of coronary atherosclerotic plaques. Coronary artery ectasia (CAE) is a rare phenotype of cardiovascular disease that may promote thrombosis and inflammatory responses leading to myocardial infarction due to abnormal dilatation of blood vessels and coronary blood flow disorders. It is a complicated disease and shows interaction between genetic and environmental factors.

**Patient concerns:**

A 34-year-old male patient was admitted to our hospital on May 12, 2016, with complaints of chest pain for 1 hour duration.

**Diagnosis:**

Coronary angiography through the emergency medical service (EMS) system showed 100% occlusion at the first turning point of the right coronary artery (RCA), along with tumor-like expansion of the proximal segment of the RCA and the end of the left main (LM) artery. The patient was diagnosed with AMI and CAE. Three-point mutations in the *ATG16L1* gene were identified by direct sequencing.

**Interventions:**

After admission, the patient underwent emergency green channel coronary angiography and percutaneous coronary intervention (PCI) to assess and unblock the stenosis and occlusion of the RCA lumen, but no stenting was performed because the catheter could not pass the second inflection point of the RCA. Aspirin enteric-coated tablets, clopidogrel sulfate tablets, tirofiban hydrochloride, and low molecular weight heparin calcium were given as anticoagulant and antiplatelet therapy. Atorvastatin calcium tablets were used to regulate blood lipid levels. Perindopril and spironolactone were used to inhibit the renin-angiotensin-aldosterone system (RAAS) to reverse myocardial remodeling. Acetylcholinesterase inhibitors (ACEI) and beta blockers were administered to resist ventricular remodeling and improve cardiac function and prognosis after the patient's blood pressure and heart rhythm were stabilized.

**Outcomes:**

After active rescue treatment, the patient recovered and was discharged. A coronary angiogram performed 2 years later showed that the RCA blood flow was restored, and the patient had recovered well.

**Conclusion:**

Three-point mutations in the *ATG16L1* gene were identified in a patient with AMI and CAE, which extended the mutation spectrum of the *ATG16L1* gene. Hence, the etiology of coronary artery aneurysmal dilatation is worthy of further investigation.

## 1 Introduction

Coronary artery disease (CAD) and its acute complication, acute myocardial infarction (AMI), are the leading causes of disability and death worldwide.^[1]^ Globally, cardiovascular disease is also the main cause of increased morbidity and mortality among young people, but there are fewer associated traditional cardiovascular risk factors and a lack of relevant research.^[2]^ Coronary artery ectasia (CAE) is a rare manifestation of coronary heart disease with incidence ranging from 1.2% to 4.9%.^[3]^ It is the aneurysmal dilatation of the coronary artery and also refers to the abnormal expansion of coronary arteries to >1.5 times that of the normal coronary artery segments.^[4]^ The most common cause of CAE is atherosclerosis, localized areas of calcification and fibrosis, and the presence of large cholesterol crystals, which reduce the elasticity of the blood vessel wall and reduces its tolerance to intraluminal blood pressure, which leads to the expansion of blood vessels^[5]^ and subsequent formation of aneurysms. Abnormal dilation of the coronary artery is often accompanied by coronary artery blood flow disorder, which can enhance thrombosis and inflammation.^[6]^ Increasing evidence shows the important role of genetic susceptibility in coronary artery disease.^[7–9]^ These diseases are caused by the interaction of genetic and environmental factors. Gene dysfunction and phenotypic alteration due to genetic variation and differential expression are caused by levels of DNA modification, regulation of RNA transcription, and mRNA translation processes. Therefore, identifying mutations in susceptibility genes is crucial in understanding the pathogenesis of complex human diseases. This will also greatly help in the accurate prevention and diagnosis of disease and in providing tailored treatment.

The lysosome degradation pathway of autophagy is a catabolic process mediated by more than 30 evolutionarily conserved autophagy-related (ATG) genes, which plays an important role in the cell, tissue, and body homeostasis.^[10,11]^ The *ATG16L1* gene provides instructions for making a protein necessary for autophagy and plays an important role in regulating autophagy membrane formation.^[12]^ Autophagy also plays an important role in controlled cell death and apoptosis.^[13]^ Additionally, autophagy is critical in maintenance of organelle and cellular metabolic homeostasis and regulation of inflammation; understandably, inflammatory diseases are often associated with autophagy dysfunction.^[14]^ In this paper, we report 3 novel mutations in the *ATG16L1* gene in a young male patient with AMI and CAE.

## 2 Case presentation

The patient was a 34-year-old man who came to our hospital on May 12, 2016, with complaints of poststernal burning pain that lasted for 1 hour without relief. Physical examination and questioning revealed that the patient had chest pain lasting >1 hour which radiated to the neck, coupled with chest tightness, excessive sweating, and nausea. Emergency electrocardiography showed sinus bradycardia and ST-segment elevation in leads II, III, and aVF. This was followed by an immediate coronary angiography that showed coronary artery dilatation lesion with diffuse angiectasia, with significantly dilated vessel diameter at the dilatation site. The maximum diameter of the proximal right coronary artery (RCA) dilatation was up to 8 mm, and all 3 vessels including the left anterior descending (LAD), left circumflex (LCX), and RCA were involved. In addition, the patient's RCA showed coexisting tumor-like expansion, stenosis, and occlusion. The dilated coronary artery showed slow blood flow, and no thrombosis was found in the dilated lumen during the operation. Based on the patient's medical history, physical examination, and related auxiliary examinations, he was hospitalized for coronary heart disease, inferior wall AMI, CAE, arrhythmia, sinus bradycardia, and level I heart function (Killip classification). As the guidewire could not passthrough owing to the severe coronary artery occlusion, it was impossible to perform coronary stent implantation. The patient was hence admitted to the intracardiac intensive care unit for further monitoring and treatment. With conservative drug treatment, regular postoperative cryptogram sulfate, atorvastatin, isopropanol fumarate, the patient's condition was adequately controlled. The patient had no history of hypertension or diabetes. He had undergone left varicocele surgery 8 years ago, had a 10-year history of smoking, and consumed alcohol occasionally. The patient's father had coronary heart disease and his mother had hypertension. There was no family history of genetic disease or infectious disease.

**Figure 1:**
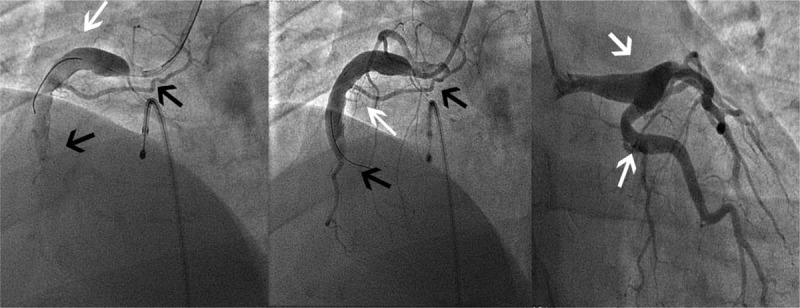
Coronary computed tomography angiography (CCTA) on admission shows coronary artery ectasias of the proximal lumen of RCA and LM terminal, and marked with a white arrow. The occlusion of the right coronary artery is indicated by the black arrow.

The results of the emergency coronary angiography performed on May 12, 2016, are presented in Figure 1. The results showed RCA was the dominant artery. The LM artery terminal showed neoplastic expansion; the lumen of the LAD was irregular without obvious stenosis or obstruction. The LCX lumen was irregular without obvious stenosis or obstruction. Aneurysmal dilatation of the proximal lumen of the RCA was observed, with 100% vessel occlusion from the first turning point. The opening occluded right coronary artery was tried. Because the guidewire could not pass through the second bend, stent implantation was abandoned. Repeated angiography showed that the distal blood flow could not be restored.

After admission on May 12, 2015, the patient underwent emergency green channel coronary angiography and percutaneous coronary intervention (PCI) to assess and dredge the stenosis and occlusion of the RCA lumen, to improve the treatment of myocardial perfusion, save the dying myocardium as much as possible, reduce the risk of death in the acute phase, and improve long-term prognosis. However, because the catheter could not pass through the second turning point of the RCA, stenting was not performed. The patient was transferred to the intensive cardiac care unit (ICCU) for conservative treatment. Aspirin enteric-coated tablets, clopidogrel sulfate tablets, tirofiban hydrochloride, and low molecular-weight heparin calcium was given for anticoagulation and antiplatelet therapy. Atorvastatin calcium tablets were used to regulate blood lipids. Perindopril and spironolactone were used to inhibit the renin-angiotensin-aldosterone system (RAAS) to reverse myocardial remodeling. Calcii Dibutyry-ladenosini Cyclophosphas for injection and Danhong injection were used to improve microcirculation and nourish the myocardium. After the blood pressure and heart rhythm were stable, ACEI and beta-blockers administered to resist ventricular remodeling and improve cardiac function and prognosis. After active rescue treatment, the patient recovered and was discharged. Three months later, the patient's condition was stable without chest pain and any other discomfort.

**Figure 2:**
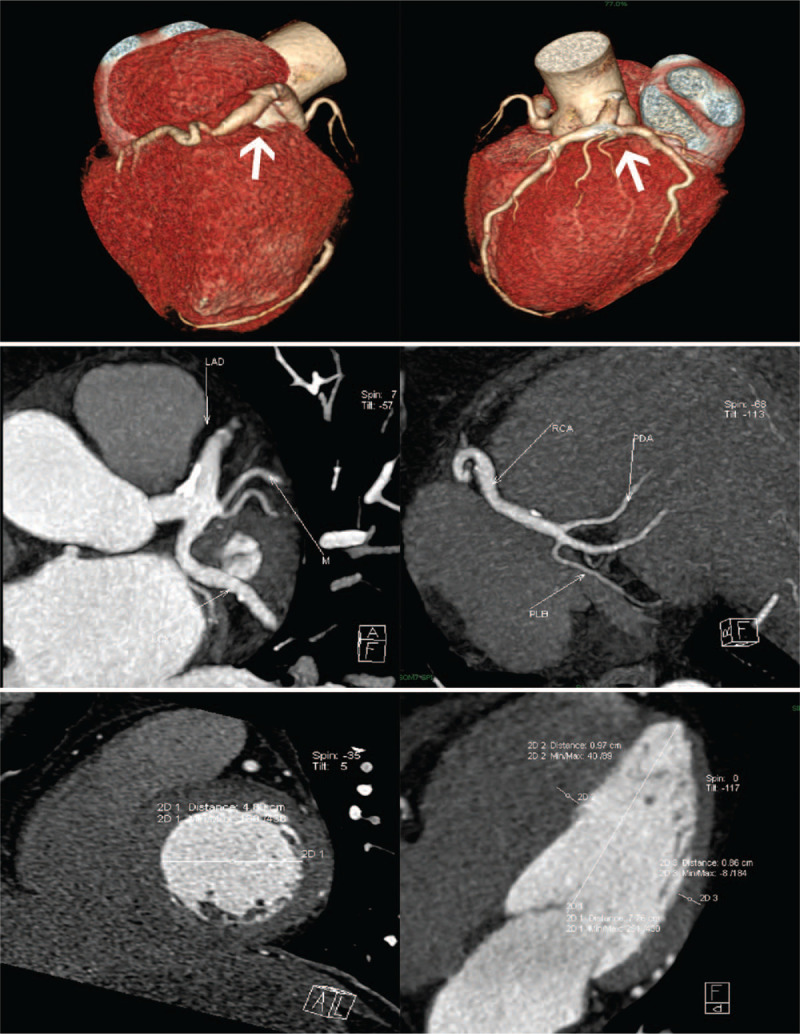
The images of the dual-source coronary computed tomography angiography (DSCT-CA) and the chest-CT. Coronary artery ectasias of the coronary artery were indicated by white arrows.

Double-source coronary computed tomography angiography (DSCT-CA) was performed on January 3, 2019. The results showed that the RCA was the dominant artery, and there was no obvious abnormality in the LCA and RCA openings (Fig. 2). The proximal part of the LAD ramus and the initial part of the LCx ramus showed local expansion. The wall of the proximal tube of the LAD branch was thickened with the formation of mixed plaques, and the stenosis rate of the adjacent lumen was 30%. The wall of the proximal segment of the left circumflex branch was thickened with mixed plaque formation, which corresponded to about 20% stenosis. Multiple non-calcified plaques and mixed plaques were found throughout the RCA, and the corresponding luminal stenosis was mild. The RCA was locally dilated at the proximal and distal ends and measured about 8-mm wide. The lumen at the second turning point of the RCA showed severe stenosis, and the lumen before and after the second turning point of the RCA expanded. An arc-like, low-density shadow was seen in the inferior wall of the left ventricle, confirming inferior wall AMI.

### 2.1 Direct DNA sequencing

The study plan was approved by the Humanistic Ethics Committee of Affiliated Hospital Jining Medical University (2016-FY-076), which strictly followed the principles of the Helsinki Declaration. The patient provided written informed consent. Peripheral whole blood samples (3 ml) were collected from the patient on July 17, 2020. Genomic DNA was extracted by using a DNA extraction kit (QIAGEN, Valencia, CA, USA) according to the manufacturer's instructions and stored at −80°C until further use. Briefly, 2000 bases upstream of the transcription start point of the *ATG16L1* gene was selected from the GenBank database (NCBI: NC_000002.12) and the PCR primers for the promoter of the *ATG16L1* gene was designed according to the sequence. The sequences of the 2 primers were ATG16L1-F: 5’-CCCAAACAAACCACAAAACC-3’ (20 bp) and ATG16L1-R: 5’-GGAGCTCACCTCCACACACT-3’ (20 bp). The polymerase chain reaction (PCR)-amplified product was a 1075 bp DNA fragment, and the primers were synthesized by Shanghai Sangon Biotech (Shanghai). The target fragment amplified by PCR was sent to Shanghai Sangon Biological Co. Ltd. for gene sequencing by the Sanger method. Chromas and DNAMAN software were used for gene sequence analysis. At the same time, the TRANSFAC database was used to carry out comparative analysis of the mutation sites in the *ATG16L1* gene promoter region, to further study the influence of gene mutations on the binding of transcription factors.

**Figure 3:**
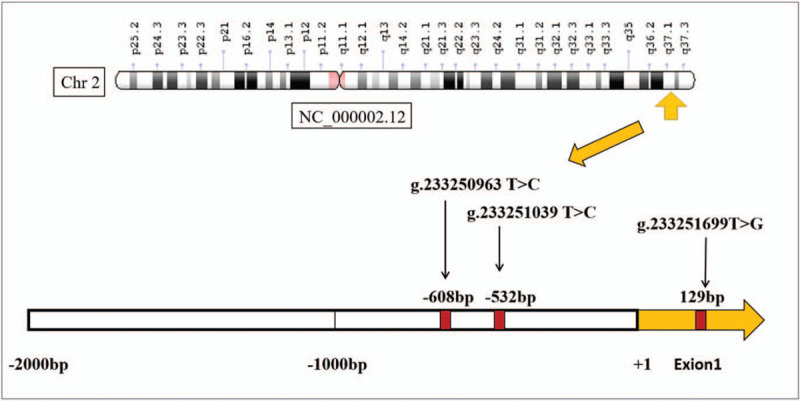
Localization of 3 SNPs in ATG16L1 genome sequence on the chromosomes (Genbank accession number NC_000002.12).

**Figure 4:**
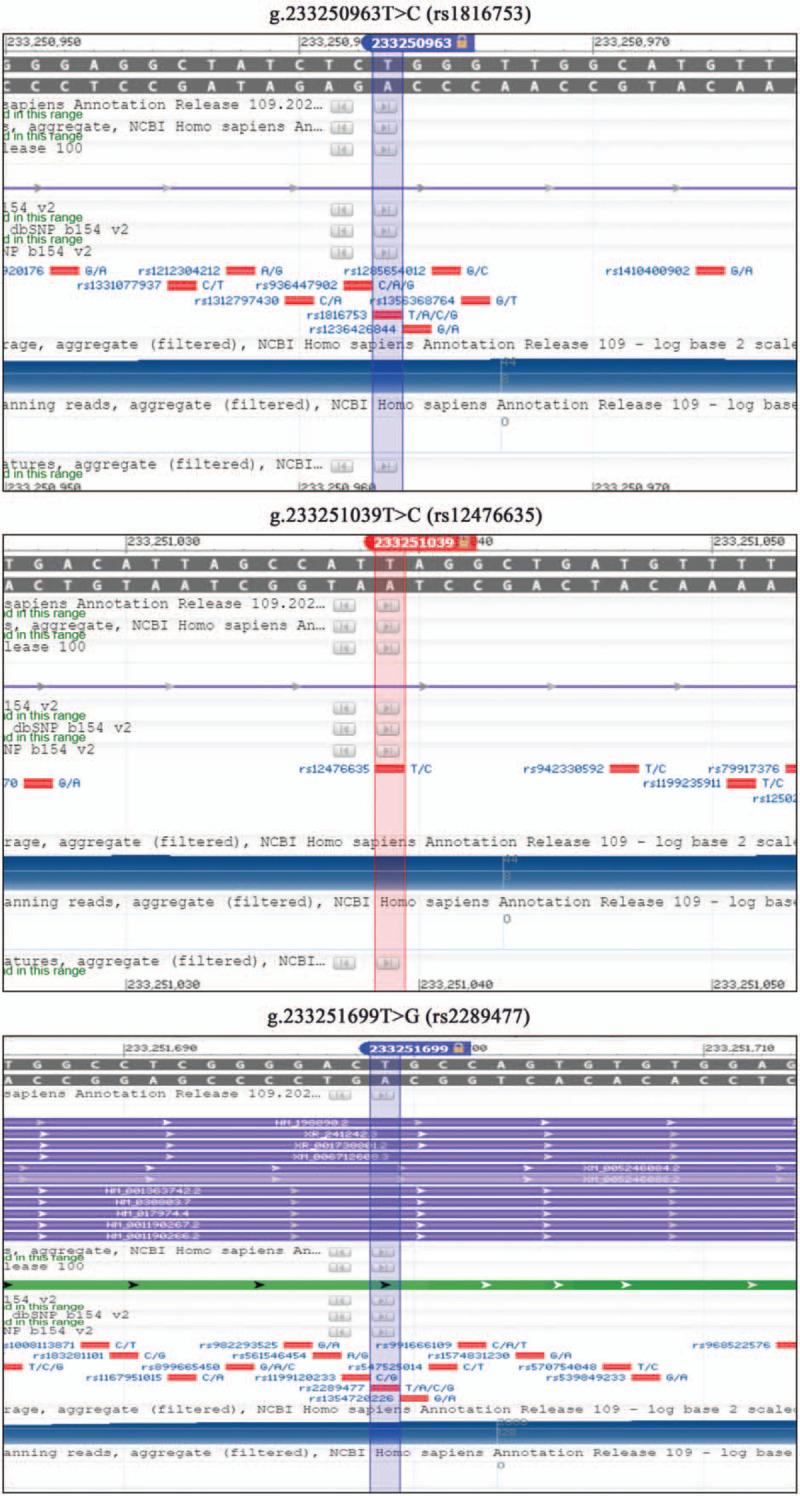
The 3 single nucleotide polymorphisms.

**Figure 5:**
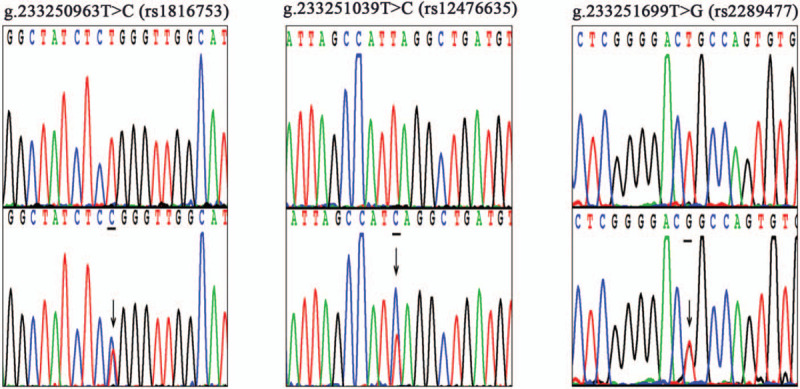
The sequencing chromatograms of 3 SNPs, and all sequences orientation of the SNPs are forward. On the uppermost layers are wild-type sequences. The bottom panels are heterozygous mutations which are marked with arrows.

After sequencing and analysis of the target gene, the results showed that there were 3 mutation sites (g.233250963T>C, g.233251039T>C, and g.233251699T>G) in the promoter region of the *ATG16L1* gene in the patient. The chromosomal locations of the 3 mutation loci are illustrated in Figure 3. Cytogenetic location: 2q37.1, which is the long (q) arm of chromosome 2 at position 37.1. In addition, after searching the NCBI database, we identified the 3 mutation sites as 3 single-nucleotide polymorphisms (SNPs) (rs1816753, rs12476635, and rs2289477) as shown in Figure 4. The gene sequencing chromatograms of these 3 mutations are shown in Figure 5. The predicted transcription factor binding sites of these mutations in *Homo sapiens* are presented in Table 1. The SNP [g.233250963T>C (rs1816753)] may create the binding sites for TRPS1 and SPDEF, and abolish the binding sites for GLI2. The SNP [g.233251039T>C (rs12476635)] may create a binding site for AP-2, and abolish the binding site for BARX2, HOX, and DLX. The SNP [g.233251699T>G (rs2289477)] may create the binding site ZNF, and abolish the binding site for NFIB, CHURC1, and HSF4. In this patient, the *ATG16L1* gene was found to have heterozygous nucleotide variations of g.233250963T>C, g.233251039T>C, and g.233251699T> g, which may lead to the binding of transcription factors to the gene promoter, thus affecting transcription level, autophagy, and possibly the occurrence and development of disease. To the best of our knowledge, the pathogenicity of these mutations has not been reported.

**Table 1:** Predicted binding sites for transcription factors affected by the SNPs in *Homo sapiens*.

SNPs	Change mode	Transcription factors
g.233250963T>C (rs1816753)	create	TRPS1, SPDEF
	abolish	GLI2
g.233251039T>C (rs12476635)	create	AP-2
	abolish	BARX2, HOX, DLX
g.233251699T>G (rs2289477)	create	ZNF
	abolish	NFIB, CHURC1, HSF4

AP-2 = Integrase-type DNA-binding superfamily protein, BARX2 = BarH-like homeobox 2, CHURC1 = churchill domain containing 1, DLX = distal-less homeobox, GLI2 = GLI family zinc finger 2, HSF4 = heat shock transcription factor 4, NFIB = nuclear factor I B, SPDEF = SAM-pointed domain-containing Ets-like factor, TRPS1 = transcriptional repressor GATA binding 1, ZNF = zinc-finger.

## 3 Discussion and conclusions

Cardiovascular diseases, a serious threat to human health, are ischemic heart diseases caused by atherosclerosis (due to intimal lipid deposition and chronic inflammation of the arteries), hypertension, hyperlipidemia, vasospasm, and embolism and are the leading cause of death worldwide.^[1,15]^ Cardiovascular disease is a common and complex disease, which is caused by the complex interaction between genetic factors and the internal and external environment.^[16]^ The traditional risk factors include hypertension, hyperlipidemia, diabetes, and obesity, which contribute to the occurrence and development of cardiovascular diseases together with genetic variants.^[17]^ Genome-wide Association Study (GWAS) has determined that 164 chromosomal loci are associated with the risk of coronary artery disease, and these variants can affect the risk of coronary heart disease.^[18]^ Almost all of the risk variants identified by GWAS are common in the population, and an average person can carry multiple risk alleles at once. Furthermore, almost all of the mutations were in the non-coding part of the genome, suggesting that these mutations are more likely to influence the regulation of gene expression, leading to disease development.^[19]^ As a core autophagy protein, ATG16L1 is involved in different stages of autophagosome biogenesis and plays a crucial role in LC3 lipidation and autophagosome maturation. The coiled-coil domain (CCD) residue of ATG16L1 mediates direct binding to phosphoinositol, which is necessary to regulate the pre-recruitment autophagy structure (PAS) for autophagy activity.^[20]^ Apart from playing a significant role in autophagy, ATG16L1 also plays a regulatory role in other aspects. The combination of lysine methylated ATG16L1 and ATG12-ATG5 conjugate can lead to autophagy inhibition and increased apoptosis of cardiomyocytes treated with ischemia-reperfusion H/R. Studies have shown that the combination of lysine-methylated ATG16L1 and ATG12-ATG5 conjugate can lead to autophagy inhibition and increased apoptosis of cardiomyocytes exposed to hypoxia/reoxygenation (H/R).^[21]^ Recent studies have shown that ATG16L1 is highly expressed in phagocytes, endothelial cells, and foam smooth muscle cells in human atherosclerotic plaques, and the expression of ATG16L1 is related to the level of proinflammatory cytokines. In the plaques of vulnerable mouse models, ATG16L1 and smooth muscle cells were strongly co-localized in early atherosclerotic lesions. In the procession of using oxidized low-density lipoprotein to promote the formation of foam cells by human macrophages, the expression of ATG16L1 and autophagy flux increased significantly. Therefore, the expression of ATG16L1 is associated with foam cell formation and plaque inflammation and may promote plaque vulnerability in the early stage of atherosclerosis.^[22]^ These results suggest that the dysfunction of autophagy and ATG16L1 may be related to the occurrence and development of the cardiovascular disease. However, the specific mechanism needs further research and exploration, and more clinical studies are needed to verify.

The risk factors of CAE are similar to atherosclerosis and other cardiovascular diseases, but not in all patients, and the actual etiology of CAE is still unknown.^[23]^ However, there is evidence that CAE is associated with genetic susceptibility, common risk factors for coronary artery disease, and abnormal vessel wall metabolism.^[24]^ In this case report, 3 mutation sites were identified in the promoter region of the *ATG16L1* gene. This may lead to changes in the binding sites of transcription factors, thus affecting the autophagy function and possibly leading to the occurrence and development of disease. With the development of CT and MRA, diagnosis and evaluation have become easier and more convenient. At present, treatment is mainly targeted towards common cardiovascular risk factors. In this case, the largest tumor-like expansion diameter measured 8 mm (<10 mm), and there was no thrombosis on the expanded lesion; therefore, there was no need to perform surgery and instead, medical treatment was adopted. The patient had multiple risk factors for thrombosis, such as giant tumor like expansion, diffuse lesion site, slow blood flow, coronary artery stenosis, and occlusion. We chose aspirin, clopidogrel bisulfate tablets, tirofiban hydrochloride, and low molecular-weight heparin calcium for anticoagulation and antiplatelet therapy. The patients were followed-up for 3 months after discharge, and no obvious discomfort was reported. However, going by clinical experience, prevention, detection, and treatment should be considered equally important and addressed to control disease development and progression. Advanced precision medicine for precise prevention, combined with preventive treatment can effectively reduce the incidence of disease and the proportion of adverse outcomes.

Advances in genetics have had a significant impact on the clinical diagnosis and treatment. Genomic testing has brought greater opportunities for diagnosis or prediction of future diagnosis, as well as increased opportunities for uncertain or accidental discoveries. Precision medicine is intended to assess the risks of specific genetic variants and disease characteristics associated with these human components and to provide personalized prevention, diagnosis, and treatment methods for them. With the advent of genome-wide association studies (GWAS), large-scale gene sequencing and functional studies have promoted a better understanding of causal risk factors, as well as clarifying the potential biological relationships and development of new therapies. At present, cardiovascular genomic medicine is dedicated to identifying and understanding heart diseases caused by genetic and genomic mechanisms. In this study, we report 3 SNPs (rs1816753, rs12476635, and rs2289477) that were identified in the promoter region of the *ATG16L1* gene of our patient with myocardial infarction and coronary artery ectasia. These genetic variations may cause gene dysfunction by altering the binding of transcription factors to affect transcription levels and translation, which may lead to protein dysfunction and changes in coronary phenotypes in the patient, leading to the occurrence and development of AMI and coronary dilation. Therefore, mutations in susceptible genes play a vital role in the development and progression of human diseases. This case report, therefore, not only provides better understanding of the relationship between genes and human disease phenotypes but also provides a genetic basis for future research on molecular mechanisms of gene dysfunction. The discovery of this new locus marks the potential beginning of a more arduous effort to understand the biological and clinical relevance of the locus, the molecular mechanism of genotypes for connecting phenotypes, and essentially increases our understanding of cardiovascular disease. This will also drive the future development of genetic testing to identify subsets of patients at high risk of cardiovascular disease and adopt the most effective treatment or preventive measures for them, thereby achieving precision medicine.

## Acknowledgments

The authors thank the patient for his consent to publish his case and the related pictures.

## Author contributions

**Conceptualization:** Bo Yan.

**Data curation:** Falan Han.

**Formal analysis:** Falan Han, Bo Yan.

**Funding acquisition:** Bo Yan.

**Investigation:** Falan Han, Bo Yan.

**Methodology:** Falan Han.

**Project administration:** Bo Yan.

**Supervision:** Bo Yan.

**Validation:** Bo Yan.

**Visualization:** Bo Yan.

**Writing – original draft:** Falan Han.

**Writing – review & editing:** Bo Yan.

## Abbreviations

Abbreviations: AMI = acute myocardial infarction, ATG16L1 = autophagy related 16 like 1, CAD = coronary artery disease, CAE = coronary artery ectasia, GWAS = genome-wide association studies, RAAS = renin angiotensin aldosterone system, SNPs = single nucleotide polymorphisms.
